# Baroreflex contribution to blood pressure and heart rate oscillations: time scales, time-variant characteristics and nonlinearities

**DOI:** 10.1098/rsta.2008.0274

**Published:** 2009-02-27

**Authors:** M. Di Rienzo, G. Parati, A. Radaelli, P. Castiglioni

**Affiliations:** 1 Biomedical Technology Department, Fondazione Don Carlo Gnocchi ONLUSVia Capecelatro 66, 20148 Milano, Italy; 2 Universita' Milano Bicocca and Istituto Auxologico Italiano20145 Milano, Italy; 3 Ospedale San Gerardo Monza20052 Milano, Italy

**Keywords:** arterial baroreflex, cardiovascular control, heart rate variability, blood pressure variability

## Abstract

The aim of this paper is to highlight the aspects of the baroreflex control of the cardiovascular system that could be relevant to the analysis and modelling of cardiovascular oscillations and regulation. In particular, complex and/or controversial issues of the baroreflex control are addressed on the basis of results obtained in previous studies by others as well as by our group. Attention has been focused on time-variant and nonlinear characteristics of the baroreflex function and on the influence of this physiological mechanism on different frequency regions of blood pressure and heart rate spectra.

## 1. Introduction

Over the centuries, the understanding of the oscillations of cardiovascular parameters (blood pressure (BP), heart rate (HR), blood flow, heart contractility, etc.) has been a puzzling issue for philosophers, clinicians and researchers. The knowledge of the mechanisms producing these oscillations is not only relevant for a better comprehension of human physiology but it may also have a major clinical impact. Indeed, for BP, it is known that an excessive variability represents a major risk factor for the occurrence of fatal events (Tozawa *et al*. 1999; Mancia & Parati 2003). On the contrary, for HR it is the reduced variability that represents a bad prognostic sign since it has been associated with a dysfunction of the autonomic mechanisms controlling the heart (Eckberg *et al*. 1971; Wolf *et al*. 1978; Bigger *et al*. 1992).

Variability of cardiovascular signals is actually due to a summation of regular rhythms and irregular fluctuations with different time scales. While it is apparent that the fastest rhythm is due to the heart contraction, the origin of the slower fluctuations may be related to a variety of factors, including neural and humoral mechanisms.

The arterial baroreflex, in its role of BP regulator, is a major player in the production of BP and HR oscillations with periods from seconds to minutes. Its involvement in the production of oscillations having periods of the order of hours or longer is still debated (Barrett & Malpas 2005; Thrasher 2005).

The aim of this paper is to highlight, in a schematic way, the aspects of the baroreflex control that could be relevant to the analysis and modelling of cardiovascular oscillations and regulation.

In particular, some complex and/or controversial issues of the baroreflex control have been addressed on the basis of available literature and the results obtained in previous studies by our group. Attention has been focused on time-variant and nonlinear characteristics of the baroreflex function and on the influence of this control mechanism on different frequency regions of BP and HR spectra.

## 2. The baroreflex function

The ultimate goal of the arterial baroreflex is to maintain BP homeostasis. This reflex mechanism counteracts deviations of BP from a reference set point by modulating HR, peripheral vascular tone and other cardiovascular variables through autonomic paths. BP information is sensed by stretch receptors (baroreceptors) mainly located on the wall of carotid arteries and aorta. When a BP change occurs, a modification in the dilation of arterial walls is sensed by these receptors and information is sent to control centres located in the brain stem through afferent neural fibres. These centres process the baroreceptor inputs and modulate autonomic outflow so as to produce changes in the cardiovascular variables (mainly HR, heart contractility and vasoconstriction) required to guarantee a proper control of BP.

Owing to its primary role in cardiovascular control, an impairment of the baroreflex may result in a significant dysregulation of BP, leading to an increased BP variability, including sudden pressure drops on shifting from supine to standing position as well as aberrant pressure rises with a major risk of fatal events such as myocardial infarction and stroke.

Although the most effective limb of the baroreflex cardiovascular control is the regulation of peripheral resistance (obtained by an efferent sympathetic modulation of the lumen of the arterioles), the overall efficiency of the baroreflex is commonly inferred from the evaluation of the baroreflex control of HR, because of its simpler assessment. In the following sections, only this limb of the baroreflex function will be considered.

A large number of techniques have been developed for the assessment of baroreflex function in a laboratory setting and in daily life. A description of these techniques is beyond the scope of this paper, but comprehensive reviews on this topic can be found in Parati *et al*. (2000) and Laude *et al*. (2004).

### (a) Complexity in the baroreflex control of cardiovascular system

Baroreflex control of circulation is actually a complex physiological function characterized by time-variant properties and nonlinear features.

A major time-variant property refers to the gain of the baroreflex control (Smith *et al*. 1969; Eckberg 1977). Indeed, it has been repeatedly reported that the magnitude of this parameter is continuously modulated over time as a result of central influences (commands from higher brain centres) aimed at optimizing the response of the cardiovascular system to the daily life challenges (Parati *et al*. 1988). For instance, during physical exercise, BP is physiologically requested to rise from its reference level in order to facilitate perfusion of muscular districts. Given the intrinsic high efficiency of the baroreflex, this pressure rise can be obtained only through a reduction in the baroreflex gain. Indeed, should the baroreflex gain remain stable at its maximal value, no pressure increase would be allowed (Wesseling & Settels 1985).

As will be discussed in §2*b*, the time modulation of the gain of the baroreflex may be a determinant of long-term BP changes.

**Figure 1:**
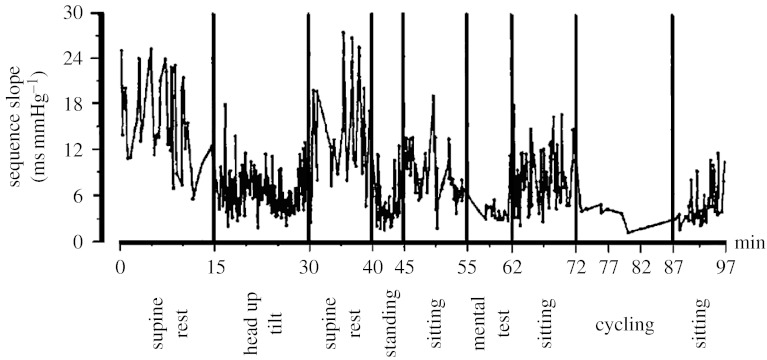
Time modulation of BRS in a subject while undergoing a series of different activities. BRS was estimated by the sequence technique. Adapted from Di Rienzo *et al*. (1997).

**Figure 2:**
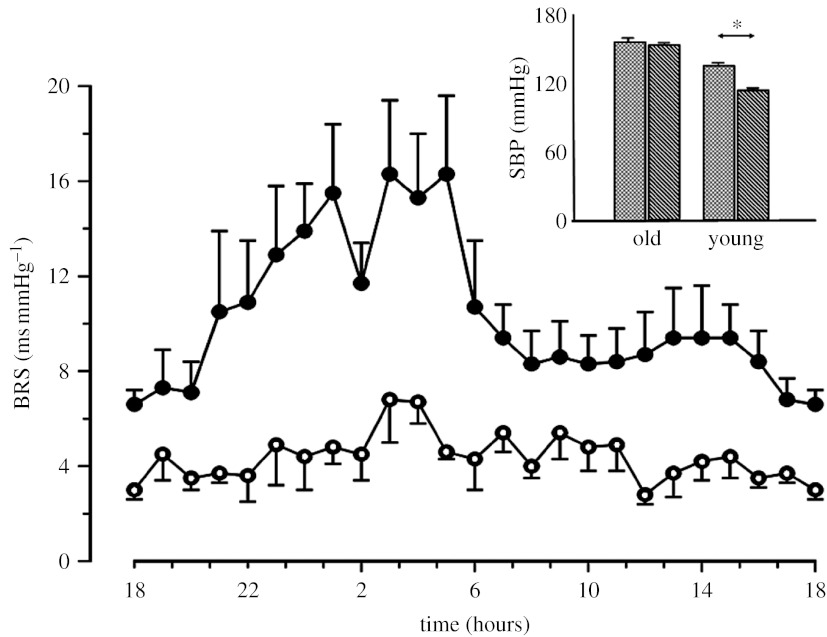
The 24 hours profile of BRS as estimated by the sequence technique in a group of young (*n*=8; filled circles) and elderly subjects (*n*=8; open circles). Inset: average SBP values observed during day and night for the two groups of subjects (stippled bars, day; left-hatched bars, night). ^*^p<0.05. Adapted from Parati *et al*. (1995*b*).

Examples of modulation of the gain of the baroreflex control of HR, hereafter also termed cardiac baroreflex sensitivity (BRS), are shown in figure 1 (in response to a series of different physical challenges) and in figure 2 (during 24 hour spontaneous behaviour). BRS is defined as the change in the RR interval (RRI, the reciprocal of HR, measured as the time interval between consecutive R peaks of the electrocardiogram) following a unitary change in BP and is expressed in ms mm Hg^−1^. From figure 1 it appears that BRS values not only depend on the different physiological conditions, but are also characterized by an ‘intrinsic’ fast variability occurring within each condition. At the moment, it is not known whether this is a true physiological phenomenon or whether it represents noise introduced by the techniques used for estimating BRS. This is an intriguing aspect of baroreflex function of interest for future research.

The baroreflex control of circulation is also characterized by several nonlinear features. The first nonlinear characteristic refers to the sensing mechanism through which the baroreceptors detect arterial pressure. As mentioned earlier, baroreceptors are stretch sensors and are located over elastic tissues (the arterial walls). Thus, the relationship between BP and baroreceptor stimulation is largely nonlinear. Moreover, the baroreceptors are differently sensitive to steady BP levels and to the rate of pressure changes with an increase in the firing rate in correspondence with pressure rises (Landgren 1952; Karemaker & Wesseling 2008). Finally, differences have been observed between the neural afferent outflow of the baroreceptors located at the carotid and aortic sites. How information stemming from these two families of baroreceptors is integrated at the central level is not entirely clear, although evidence of nonlinear interactions has been produced (Brunner *et al*. 1984; Bertinieri *et al*. 1987).

**Figure 3:**
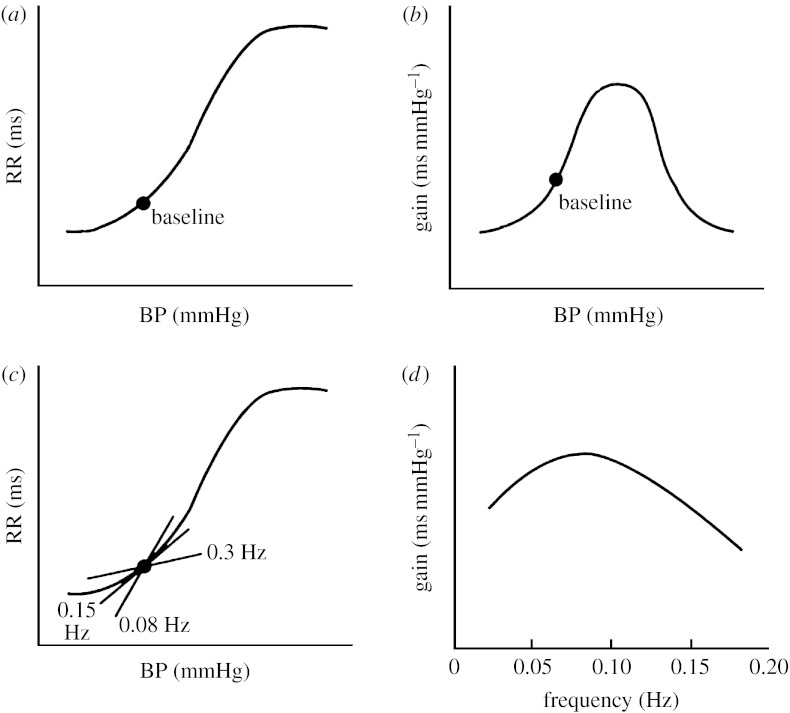
Schematic of different features of the gain of the baroreflex control of HR. (*a*,*b*) Static (steady-state) gain, (*c*,*d*) dynamic gain. (*a*) Sigmoidal curve describing the relationship between static BP values and the reflex RRI responses. (*b*) Gain (estimated as the local slope of the sigmoidal curve) versus static BP values. (*c*) Changes in the slope of the sigmoidal curve as a function of the BP frequency. (*d*) Example of change in the baroreflex gain as a function of the BP frequency at a given BP average value. Adapted from Parati *et al*. (1995*a*).

Also the relationship between the reflex RRI value changes produced by the baroreflex in response to different BP values is nonlinear. Indeed, this relationship can be usually represented by a sigmoidal curve and includes a threshold, a saturation point and, in between, a linear zone (figure 3). Only BP variations occurring in the central linear part of the sigmoid curve produce a marked reflex RRI response, while comparable changes occurring at both the extremes of the curve produce only a more limited RRI response (Mancia & Mark 1983). For any stable BP value, the gain (BRS) is estimated as the slope at that value on the sigmoid curve. It should be recalled, however, that if BP fluctuates, BRS may change as a function of the frequency of the BP oscillation (Parati *et al*. 1995*a*).

More recently, experimental data have suggested that the baroreflex function may also be influenced by a stochastic resonance phenomenon (Hidaka *et al*. 2000; Soma *et al*. 2003). Stochastic resonance is a phenomenon that occurs in nonlinear systems and which allows the system to improve its capability to detect small signals, when a limited amount of noise is added. It may occur in physiological systems with a sensory threshold. In this case, the addition of a moderate amount of noise to a subthreshold input signal allows the signal to cross the detection threshold. In neurosciences, this phenomenon was observed in a variety of neuronal systems for the transmission of weak signals. The presence of stochastic resonance in the baroreflex has been demonstrated by injecting a small amount of noise on carotid baroreceptors through a pneumatic neck chamber and by observing an augmented sensitivity of the baroreflex response to an external subthreshold stimulus obtained by an oscillating tilt table (Hidaka *et al*. 2000).

Another aspect making the baroreflex physiology complex is the resetting phenomenon (McCubbin *et al*. 1956; Seagard *et al*. 1992). Similar to other biological receptors, baroreceptors also tend to reduce their firing rate and update their operating point to the new average level of stimulation when subjected to a sustained stimulation for a long period (hours, days and months). It has been suggested that this phenomenon may play a role in the occurrence of hypertension (Krieger 1986). A review on this topic may be found in Thrasher (2005).

Finally, it should be considered that the baroreflex control of cardiovascular variables aimed at counteracting BP changes is not always completely effective. Indeed, these cardiovascular variables are also under the influence of other reflexes or mechanisms, which may partially or totally mask, contrast, counteract the baroreflex drive (e.g. central influences, humoral factors, cardiopulmonary reflex, chemoreflex, metaboreflex; Somers *et al*. 1991; Du & Chen 2007; Iellamo *et al*. 2007) or may additively contribute to the baroreflex output (e.g. Ray 2000; Di Rienzo *et al*. 2008). Peripheral somatosensory inputs have also been suggested to influence the baroreflex function. Indeed, it was reported that activation of skeletal muscle afferent fibres, by either muscle contraction or stretch, may increase the carotid sinus threshold pressure of the baroreceptors, so as to limit the baroreflex efficiency and facilitate the BP and HR rise required by physical exercise (Mancia *et al*. 1982; Potts & Mitchell 1998).

The presence of interferences on the baroreflex control of HR can be simply verified by observing that, in healthy subjects, spontaneous and progressive beat-to-beat increases or decreases (ramps) in systolic blood pressure (SBP) are not always accompanied by the expected baroreflex-driven lengthening or shortening in RRI. In order to quantify this phenomenon, we previously proposed a specific index, the baroreflex effectiveness index (BEI), defined as the ratio between the number of spontaneous SBP ramps followed by a reflex RRI change and the total number of SBP ramps observed in a given time window (Di Rienzo *et al*. 2001). We found that in healthy humans, the 24 hours average BEI value was approximately 0.21, with a marked day–night modulation. These data suggest that in normal subjects, arterial baroreflex is effective in producing reflex RRI changes in response to only a fraction of all SBP ramps, possibly because of central inhibitory influences or interferences at sinus node level by non-baroreflex mechanisms.

### (b) Models of the baroreflex

In the last few decades, a large number of mathematical models have been proposed to better clarify the functioning of the baroreflex and its interactions with other control mechanisms. Differences among the proposed models concern the approach used (black box or based on physiological descriptions), how the aspects of the control are simulated, the level of details by which nonlinearities are modelled, the type of variables considered (continuous or pulsatile variables), and the capability to include specific features of real cardiovascular dynamics, such as respiratory entrainment, the 10 s rhythm or long-term BP fluctuations.

The first mathematical models of the baroreflex were based on detailed physiological descriptions of the heart and vasculature (Beneken & De Wit 1967; Guyton *et al*. 1972); however, they were unable to reproduce specific features of cardiovascular dynamics, such as the spontaneous variability of HR and BP. A significant step forward in this direction was represented by the model proposed by Hyndman *et al*. (1971), which could simulate the genesis of a spontaneous 10 s rhythm in BP by including a nonlinear element followed by a time delay in the baroreflex feedback. This baroreflex model was further developed to explain also the phenomenon of entrainment between respiratory sinus arrhythmia and the 10 s rhythm (Kitney 1979). The concept of a nonlinear element with a delay in the baroreflex feedback was refined and deeply analysed in other studies to obtain simulations more similar to real signals (Cavalcanti & Belardinelli 1996; Ringwood & Malpas 2001).

A different approach was followed by Wesseling & Settels (1985), which proposed a model to explain the so-called ‘baroreflex paradox’, i.e. how large BP oscillations can occur in real life in spite of the fast and efficient action of the baroreflex. As mentioned earlier, the paradox can be explained by considering the modulation of the baroreflex gain: when BP is requested to increase, central influences reduce BRS and thus the efficiency of the baroreflex control. On this premise, the authors postulated that also very low BP fluctuations can be explained by a proper modulation of the baroreflex gain, in their model simulated by ‘1/*f*’ noise. They could also simulate a 10 s rhythm as a resonance owing to the delay in peripheral resistances. Their model was not aimed at considering the pulsatile nature of arterial pressure, and continuous variables were used.

By contrast, the simulations of the beat-by-beat relationships between cardiovascular variables (heart interval, systolic and diastolic BPs) were the aim of the model proposed by De Boer *et al*. (1987). This model was based on a system of differential equations representing the baroreflex control on HR and peripheral resistance, the Windkessel properties of the arterial tree, the contractile properties of the myocardium and the effects of respiration on BP. Also in this model, the 10 s rhythm was explained as a resonance due to a delay in the sympathetic loop. The accurate simulation of the relationships between beat-by-beat variables was also the aim of a model proposed by TenVoorde & Kingma (2000). This model consisted of a beat-to-beat haemodynamic part linked to a continuous-modelled neural control part: the link between continuous and beat-to-beat components was obtained through an integral pulse frequency modulator block acting as cardiac pacemaker, and driven by sympathetic and vagal outflows.

A model of the baroreflex was also proposed by Seidel & Herzel (1998). Their focus was on simulating, through a set of differential equations, the nonlinear coupling between the cardiac pacemaker and the baroreflex control loop and the interactions among the pulse oscillation, respiration and Mayer waves. Their nonlinear model explained the genesis of sustained oscillations induced by sympathetic delays in terms of a Hopf bifurcation, and the occurrence of more complex rhythms, including entrainment and chaotic dynamics, following increases in BRS.

Other models have been proposed to specifically investigate the BP–RRI closed-loop relationship as a function of the baroreflex control during spontaneous behaviour (Appel *et al*. 1989; Baselli *et al*. 1994; Patton *et al*. 1996; Wang & Chon 2007). These models aim at disentangling the crucial issue of causality between BP and RRI, i.e. they try to determine how much of the link between these variables is owing to (i) the direct action of BP on RRI (through the baroreflex), (ii) the feed-forward mechanical action of RRI on BP (through the change in cardiac output) or (iii) the possible factors that simultaneously influence BP and RRI (e.g. central drives, respiration, the baroreflex itself that simultaneously controls peripheral resistances and RRI). Actually, most of the approaches currently used for the analysis of spontaneous BP–RRI interactions (including coherence analysis and most of the techniques for the assessment of spontaneous BRS) are based on an open-loop description of the BP–RRI relationship and do not consider causality. It has been suggested that these common but simplistic approaches disregard most physiological influences and tend to attribute all observed BP and RRI changes solely to the arterial baroreflex (Porta *et al*. 2000, 2002).

Models taking into account causality have proven to be able to provide insights into cardiovascular control (Nollo *et al*. 2005). However, further studies are still required to fully understand the potentiality of this approach and make it applicable in clinical practice. Further details on causality may be found in the papers by Batzel *et al*. (2009) and Nollo *et al*. (2009) published in this issue of the *Philosophical Transactions A*.

### (c) Time scales of the baroreflex action on HR and BP variability

After having considered nonlinearities, resetting phenomena and interactions from other mechanisms, one might wonder what is the real zone of influence of the baroreflex on HR variability (HRV) and BP variability (BPV) or, in other words, what is the real time scale of the baroreflex control action.

In this regard, a large number of studies have been performed on humans and animals to investigate the baroreflex effects on HR and BP oscillations with periods from seconds to minutes. The baroreflex effects on the slowest components of variability (tens of minutes, hours and days) have been much less frequently addressed and most of the studies in this area were based on the analysis of the long-term effects on BPV and HRV of surgical baroreceptor denervation in animals. This experimental procedure, often termed sinoaortic denervation (SAD), is based on the surgical severing of the afferent neural fibres stemming from the baroreceptors in the carotid and aortic areas. In practice, SAD opens the baroreflex loop. Experiments based on SAD provide a deep insight into the cardiovascular control, but three aspects need to be considered for a correct interpretation of results. First, in different animal species, it is possible that the same afferent neural fibres conveying baroreceptor information also convey information provided by other receptors, e.g. chemoreceptors. The surgical procedure would thus cut all these connections simultaneously. Second, the extrapolation of animal data to human baroreflex control patterns should be done with caution, by taking into account the physiological differences between humans and the various animal species. Third, when the focus is on long-term effects of denervation, it should be considered that after a while other control mechanisms may intervene to vicariate baroreflex control. In this view, the net difference in BPV and HRV between what is observed in intact condition and a long period after the removal of the baroreflex action (by denervation) might be indicative not only of the baroreflex action but also of the effects of other mechanisms possibly intervening after SAD.

### (d) Baroreflex versus BPV: ultra-low frequencies (hours and days)

The effect of baroreflex on BP oscillations with periods of hours and days is still a matter of debate with controversial findings and unexplored issues. Moreover, differences in the animal species investigated, the experimental procedure applied and the techniques used for data analysis make it difficult to put all results in a coherent framework.

In this section we will try to briefly summarize some of the key points in this area. The pioneering study in this field was by Cowley *et al*. (1973). In this study, BPV was investigated in dogs before and several days after SAD. Data analysis showed that after denervation BP mean value was only minimally increased while fast BPV dramatically increased in response to postural changes and physical activity. From these results, it was concluded that the baroreflex has no influence on the long-term component of BP (as reflected by the invariance in mean value) while it plays a role in buffering the fastest components of BPV (as estimated by the increased standard deviation after denervation).

Over time, other studies addressed this issue through experiments in animals. The results were diverse and in some cases it was concluded in favour of (Thrasher 2002; Lohmeier *et al*. 2004) and in other cases against (see review in Cowley 1992) the hypothesis of a baroreflex involvement in the genesis of the slowest components of BPV.

**Figure 4:**
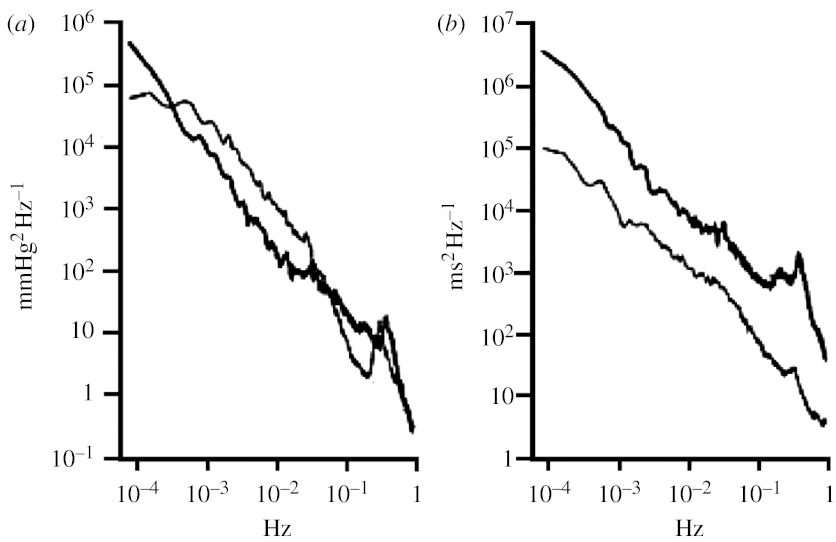
Average broadband power spectra of (*a*) SBP and (*b*) PI in a group of eight cats before (thick line) and after (thin line) SAD. Adapted from Di Rienzo *et al*. (1996).

Our group previously investigated this issue by evaluating the spectral changes in BPV and HRV observed in unanaesthetized cats before and 7–10 days after SAD (Di Rienzo *et al*. 1991, 1996). Each data recording lasted more than 3 hours and a single broadband spectrum was estimated from the whole recording in order to evaluate the power of systolic and diastolic BP and of heart rhythm oscillations with periods from approximately 1 s to 3 hours. In this study, the heart rhythm was estimated by the pulse interval, PI, i.e. the time distance between two consecutive systolic BP peaks. The average SBP and PI broadband spectra for the animals in intact and after SAD condition are illustrated in figure 4. It is apparent that all SBP spectral components are actually modified by denervation, with differentiated effects over the frequency axis, including frequency regions where the removal of baroreflex function produced a power increase (thus indicating a baroreflex buffering action before SAD) and regions where the spectral power was reduced (indicating a baroreflex pro-oscillatory action before SAD).

In particular, as far as the ultra-low frequencies are concerned, our data provided evidence of a significant BP power reduction for oscillations having a period longer than 1600 s, thus indicating that in intact animals the baroreflex does not buffer but rather paradoxically enhances the amplitude of these BP oscillations.

**Figure 5:**
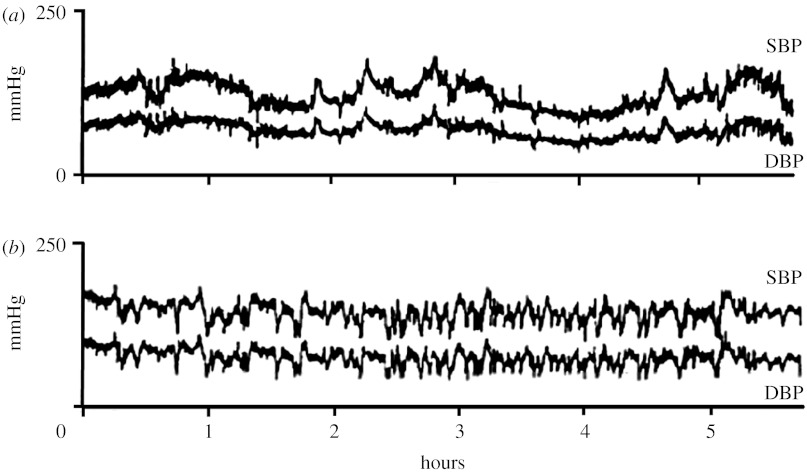
Beat-by-beat time profile of systolic and diastolic BP from a cat (*a*) before and (*b*) after SAD. Adapted from Castiglioni (1993).

The disappearance of these ultra-low fluctuations of BP after SAD is also apparent by a visual inspection of the BP recordings before and after denervation (figure 5).

As mentioned above, it has been suggested that one of the mechanisms through which the baroreflex might influence ultra-low components of BPV is the change in the baroreflex gain over time. With the support of a mathematical model, Wesseling & Settels (1985; see §2*c*) suggested that not only ultra-low frequency spectral components of BPV, but also, more generally, the complete 1/*f* trend of BP spectra may be obtained by a proper modulation of the baroreflex gain. Experimental data collected by our group in a previous study indicate that the 1/*f* trend in the BP spectra estimated in humans over the 24 hours is actually accompanied by 1/*f* spectra of the baroreflex gain (Di Rienzo *et al*. 1993). In this perspective, the baroreflex might provide a contribution also in the day–night BP modulation. Indeed, it has been shown that in young healthy subjects the baroreflex gain is lower during the day and higher at night (figure 2) and the increased efficiency of the baroreflex control at night might explain the nocturnal BP reduction. The hypothesis is corroborated by the fact that in elderly hypertensive subjects the day–night BRS modulation is blunted and the nocturnal BP drop is importantly reduced (Parati *et al*. 1995*a*).

### (e) Baroreflex versus BPV: very low frequencies

Persson *et al*. (1990) reported that in dogs the power of BP fluctuations with a period of approximately 1200 s was significantly increased after SAD. Our broadband analysis of data in cats (figure 4) not only confirmed this finding but also showed that after denervation SBP and DBP power significantly increases over a wide frequency band between 0.03 Hz and 6×10^−4^ Hz, thus including oscillations with periods from 30 to 1600 s. These data indicate that in this region an intact baroreflex exerts its buffering function on BPV, possibly through adrenergic mechanisms (Radaelli *et al*. 2006*a*). It may be worth noting that this region roughly includes the very low-frequency region, as traditionally defined in HRV studies (0.003–0.04 Hz; Task Force 1996).

**Figure 6:**
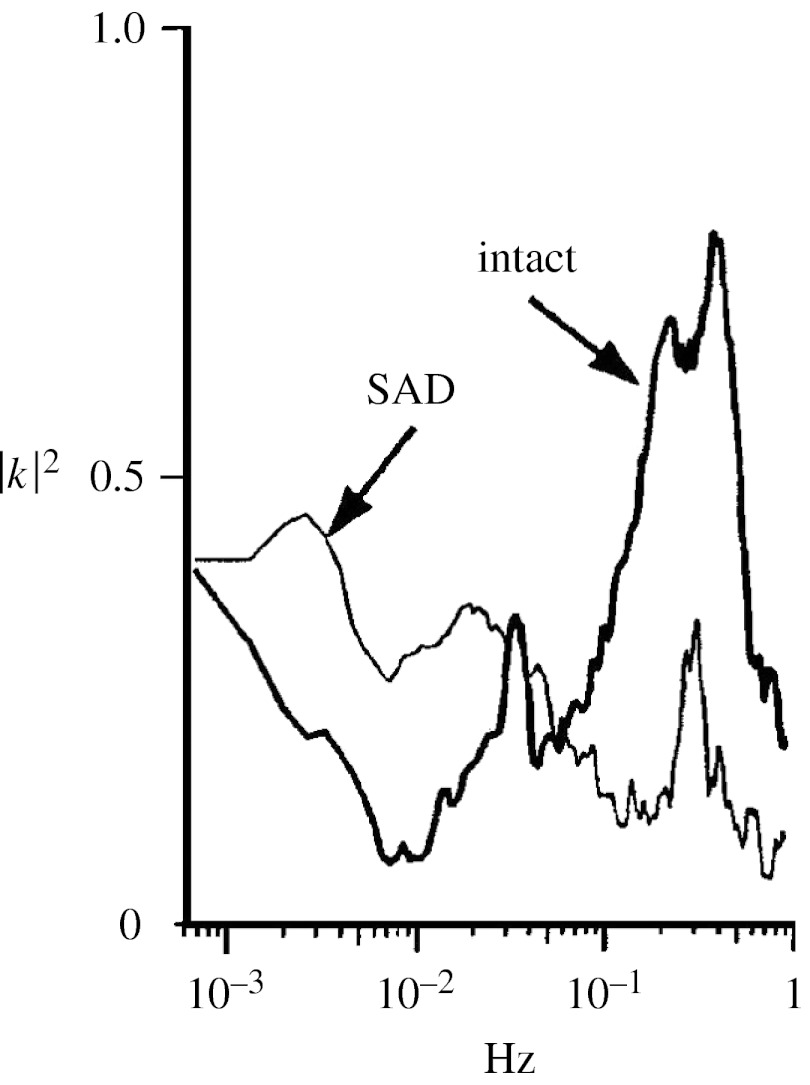
Mean squared coherence modulus between systolic BP and PI for the group of eight cats before and after SAD. Adapted from Di Rienzo *et al*. (1996).

In the above mentioned cats, the effects of SAD on the SBP–PI coherence was also evaluated (Di Rienzo *et al*. 1996). As shown in figure 6, the opening of the baroreflex produces an increase in the squared coherence modulus at frequencies lower than 0.01 Hz. Since the squared coherence modulus quantifies the strength of the linear coupling between variables, these data may suggest that in this frequency region the buffering action of the baroreflex (demonstrated by power spectral analysis) is exerted through nonlinear strategies. This might be explained by considering that at variance from the faster frequencies where the baroreflex controls only HR, in this frequency region it also directly controls BP through a modulation of peripheral resistances. Since HR and peripheral resistance controls are characterized by specific nonlinearities and time constants (Mancia & Mark 1983), their simultaneous activation might be the cause of the observed reduction in the linear link between variables. After SAD, the baroreflex influence is removed and the mechanical feed-forward PI–SBP link prevails thus increasing the linearity of their relationship.

### (f) Baroreflex versus BPV: low frequency

The low frequency (LF) band is usually defined as the region including fluctuations from 0.04 to 0.15 Hz. The main component of this frequency region is the 10 s oscillation. Concerning the genesis of this rhythm in BP, the prevalent hypothesis is that it is caused by vasomotion phenomena induced by a resonance occurring in the baroreflex loop and mediated by the sympathetic drive to the vessels (Guyton & Harris 1951; De Boer *et al*. 1987; Julien 2006). In other words, it is the intrinsic structure of the baroreflex loop that would produce the ‘10 s’ oscillation in BP. This hypothesis is supported by our data in cats. Indeed, as shown in figure 4, the power near to 0.1 Hz significantly decreased in denervated animals (Di Rienzo *et al*. 1996; Mancia *et al*. 1999).

### (g) Baroreflex versus BPV: high frequency

The high frequency (HF) region includes frequencies from 0.15 to 0.4 Hz and thus also the respiratory-driven BP oscillation (if the respiratory frequency remains within the normal limits). According to the prevalent hypothesis, respiratory activity directly influences BP through changes in the intra-thoracic pressure that in turn modify venous return and thus atrial filling (Dornhorst *et al*. 1952; De Boer *et al*. 1987; Sleight *et al*. 1995). In agreement with this reasoning, actually we did not observe any change in the high-frequency BP power after denervation (figure 4). However, as described in §2*h*, the baroreflex senses the BP respiratory oscillation and modulates RRI accordingly. Thus, the observed invariance of the BP power after denervation not only indicates that the baroreflex is not involved in the genesis of this oscillation, but also that the baroreflex modulation of HR is ineffective in buffering HF BP oscillations (Mancia *et al*. 1999).

The mechanical hypothesis of the respiratory oscillation of BP is supported also by the experimental evidence that when the baroreflex is deactivated, for example after brain death, high-frequency respiratory oscillations induced by the mechanical ventilator are still present in BP, while they virtually disappear in HR (Conci *et al*. 2001).

### (h) Baroreflex versus HR variability

In our denervated cats, removal of the baroreflex function resulted in a significant drastic reduction in HR power over the whole frequency range addressed in our study, i.e. from seconds to hours (figure 4*b*).

Some further consideration can be made by comparing this result with the effects of denervation on BPV. In particular, the drastic HR power reduction at 0.1 Hz after SAD supports the hypothesis that most of the power in this band observed in physiological condition is actually due to the attempt of the baroreflex to buffer the BP oscillation occurring at the same frequency and possibly caused by a resonance phenomenon as mentioned earlier (Julien 2006).

This is also supported by the observation of Radaelli *et al*. (2006*b*) showing that in heart failure patients, an increase in LF oscillations of HR was concomitant with an improvement in BRS. Moreover, in paraplegic subjects with a spinal lesion below the fifth thoracic vertebra (i.e. with intact vagal outflows to the heart but impaired sympathetic heart control) a reduction in the LF power of HR was shown (Castiglioni *et al*. 2007).

These findings do not exclude the possibility of a minor contribution from a direct drive on HR from central oscillators as suggested by some authors (Montano *et al*. 1996).

In addition, the observed important HR power reduction in the respiratory frequency band after SAD provides evidence of the major role played by the baroreflex also in the production of the respiratory sinus arrhythmia. It is worth noting, however, that a small HR spectral peak still survives denervation, supporting the hypothesis of a minor direct influence on the sinus node of the respiratory-induced changes in right atrium filling. This hypothesis is in line with the observed presence of small but clear HR power peaks at the respiratory frequency in heart transplant patients (Bernardi *et al*. 1989). The transplanted heart is denervated and thus the only reasonable mechanism for explaining the residual HF peak is the direct influence of venous return to the sinus node.

In healthy subjects, it has been suggested that a contribution to the total respiratory sinus arrhythmia may also derive from a direct central drive (Eckberg & Karemaker in press).

## 3. What is the baroreflex influence on nonlinearities in the BP–RRI interaction?

While the baroreflex function is rich in nonlinearities, the nonlinear behaviour of the BP–RRI interaction cannot be taken for granted. Indeed, experimental data have shown that when an overall analysis of the BP–RRI coupling is performed by the corrected cross-conditional entropy on a time scale of minutes, a prevalence of linear components is observed in healthy subjects at rest. The overall level of linearity in the BP–RRI coupling further increases during tilt, but drastically drops in patients after myocardial infarction (Nollo *et al*. 2002). Similarly, an overall linearity was observed by applying a nonlinear vector autoregressive model (Wang & Chon 2007). However, this is not to say that BP and RRI are invariably linearly linked over all frequencies. Indeed, as mentioned earlier in relation with the data illustrated in figure 6, there is a frequency region at approximately 0.01 Hz where the baroreflex is active and exerts a link between BP and RRI, but the coherence analysis suggests that such a link could be nonlinear. This is an additional puzzling aspect of the baroreflex control worthy to be further explored.

## 4. Conclusions

An increasing number of studies emphasize the importance of the assessment of arterial baroreflex function for diagnostic and prognostic purposes. This has fuelled a growing interest in the study of this control mechanism, the development of new techniques for the evaluation of its efficiency and the design of new interpretative models aimed at clarifying unclear aspects of its control function. With this paper, we aimed at contributing to these demanding tasks, by sharing our experience and providing a schematic overview of the most important aspects of the baroreflex control.

Since a huge amount of data exist on the baroreflex physiology, we had to drastically limit the number of issues addressed in this paper and the depth of description for each issue. For space reasons, some important aspects of the baroreflex function have been completely omitted, such as the baroreflex control of peripheral resistance, the relationship between baroreflex and humoral factors, the influence of the baroreflex on renal function (and thus on the long-term pressure control by modulation of body fluids) and possible influences by genetic factors.

Moreover, when addressing topics that are still a matter of debate, we have been forced to illustrate only the prevalent hypotheses. In such cases, however, we provided bibliographic references for the reader interested in deepening his/her knowledge on the matter.

On specific topics, we also illustrated the data coming from our own work. We do hope that this mix of literature and personal experience might be of interest to scientists involved in the analysis of cardiovascular control and might foster further research in this area. Actually, the data summarized in our paper highlight a large number of issues that are still unclear and that deserve to be addressed in more depth in future studies. Among them, we mention the need for further investigations on the time scale of the baroreceptor resetting phenomenon, the interaction between baroreflex and other control mechanisms, the ‘intrinsic BRS variability’ observed also when a subject is in a steady-state condition, how baroreflex nonlinearities are reflected in nonlinearities in the BP and RRI signals, and the development of new approaches to investigate the baroreflex function by considering causality in the BP–RRI interaction.

Another area of future research concerns the development of new methods for the assessment of spontaneous baroreflex control of peripheral resistances. Indeed, at the moment, research on this important part of the baroreflex physiology is limited by the complexity of the available techniques that allow us to address this issue mainly in a laboratory setting, through the application of external stimuli to baroreceptor areas (via a neck chamber device). The few model-based approaches so far developed (e.g. Mukkamala *et al*. 2003) provided positive preliminary results and foster further research in this field.

## References

[bib1] Appel, M. L., Saul, J. P., Berger, R. D. & Cohen, R. J. 1989 Closed-loop identification of cardiovascular regulatory mechanisms. In *Proc. Computers in Cardiology, Long Beach, CA*, pp. 3–8. Washington, DC: IEEE Computer Society Press.

[bib2] Barrett C.J, Malpas S.C (2005). Problems, possibilities, and pitfalls in studying the arterial baroreflexes' influence over long-term control of blood pressure. Am. J. Physiol. Regul. Integr. Comp. Physiol.

[bib4] Baselli G (1994). A model for the assessment of heart period and arterial pressure variability interactions and of respiratory influences. Med. Biol. Eng. Comp.

[bib3] Batzel J, Baselli G, Mukkamala R, Chon K.H (2009). Modelling and disentangling physiological mechanisms: linear and nonlinear identification techniques for analysis of cardiovascular regulation. Phil. Trans. R. Soc. A.

[bib5] Beneken J.E.W, De Wit B, Reeve E.B, Guyton A.C (1967). A physical approach to hemodynamic aspects of the human cardiovascular system. Physical bases of circulatory transport.

[bib6] Bernardi L, Keller M, Sanders M, Reddy P.S, Meno F, Pinsky M.R (1989). Respiratory sinus arrhythmia in the denervated human heart. J. Appl. Physiol.

[bib7] Bertinieri G, Cavallazzi A, Jaszlitz L, Ramirez A.J, Di Rienzo M, Mancia G (1987). Differential control of blood pressure and heart rate by carotid and aortic baroreceptors in unanaesthetized cats. J. Hypertens.

[bib8] Bigger J.T, Fleiss J.L, Steinman R.C, Rolnitzky L.M, Kleiger R.E, Rottman J.N (1992). Frequency domain measures of heart period variability and mortality after myocardial infarction. Circulation.

[bib9] Brunner M.J, Greene A.S, Kallman C.H, Shoukas A.A (1984). Interaction of canine carotid sinus and aortic arch baroreflexes in the control of total peripheral resistance. Circ. Res.

[bib10] Castiglioni, P. 1993 The baroreceptors and the spontaneous variability of blood pressure and heart rate. PhD thesis.

[bib11] Castiglioni P, Di Rienzo M, Veicsteinas A, Parati G, Merati G (2007). Mechanisms of blood pressure and heart rate variability: an insight from low-level paraplegia. Am. J. Physiol. Regul. Integr. Comp. Physiol.

[bib12] Cavalcanti S, Belardinelli E (1996). Modeling of cardiovascular variability using a differential delay equation. IEEE Trans. Biomed. Eng.

[bib16] Conci F, Di Rienzo M, Castiglioni P (2001). Blood pressure and heart rate variability and baroreflex sensitivity before and after brain death. J. Neurol. Neurosurg. Psychiatry.

[bib15] Cowley A.W (1992). Long-term control of arterial blood pressure. Physiol. Rev.

[bib14] Cowley A.W, Liard J.F, Guyton A.C (1973). Role of the baroreceptor reflex in daily control of arterial pressure and other variables in dogs. Circ. Res.

[bib17] De Boer R.W, Karemaker J.M, Strackee J (1987). Hemodynamic fluctuations and baroreflex sensitivity in humans: a beat-to-beat model. Am. J. Physiol. Heart Circ. Physiol.

[bib18] Di Rienzo M, Parati G, Castiglioni P, Omboni S, Ferrari A.U, Ramirez A.J, Pedotti A, Mancia G (1991). Role of sinoaortic afferents in modulating BP and pulse interval spectral characteristics in unanesthetized cats. Am. J. Physiol. Heart Circ. Physiol.

[bib19] Di Rienzo, M., Castiglioni, P., Parati, G., Frattola, A., Mancia, G. & Pedotti, A. 1993 Effects at 24-h modulation of baroreflex sensitivity on blood pressure variability. In *Proc. Computers in Cardiology*, pp. 551–554. Washington, DC: IEEE Computer Society Press.

[bib20] Di Rienzo M, Castiglioni P, Parati G, Mancia G, Pedotti A (1996). Effects of sino-aortic denervation on spectral characteristics of blood pressure and pulse interval variability: a wide-band approach. Med. Biol. Eng. Comput.

[bib21] Di Rienzo M, Parati G, Mancia G, Pedotti A, Castiglioni P (1997). Investigating baroreflex control of circulation using signal processing techniques. IEEE EMB Mag.

[bib22] Di Rienzo M, Parati G, Castiglioni P, Tordi R, Mancia G, Pedotti A (2001). Baroreflex effectiveness index: an additional measure of baroreflex control of heart rate in daily life. Am. J. Physiol. Regul. Integr. Comp. Physiol.

[bib23] Di Rienzo M, Castiglioni P, Iellamo F, Volterrani M, Pagani M, Mancia G, Karemaker J.M, Parati G (2008). Dynamic adaptation of cardiac baroreflex sensitivity to prolonged exposure to microgravity: data from a 16-day spaceflight. J. Appl. Physiol.

[bib24] Dornhorst A.C, Howard P, Leathard G.L (1952). Respiratory variations in blood pressure. Circulation.

[bib25] Du Y.H, Chen A.F (2007). A ‘love triangle’ elicited by electrochemistry: complex interactions among cardiac sympathetic afferent, chemo-, and baroreflexes. J. Appl. Physiol.

[bib26] Eckberg D.L (1977). Baroreflex inhibition of the human sinus node: importance of stimulus intensity, duration, and rate of pressure change. J. Physiol.

[bib27] Eckberg, D. L. & Karemaker, J. M. In press. Point: counterpoint “respiratory sinus arrhythmia is due to a central mechanism vs. the baroreflex mechanism”. *J. Appl. Physiol.* (10.1152/japplphysiol.91107.2008) 18719228

[bib28] Eckberg D.L, Drabinski M, Braumwald E (1971). Defective cardiac parasympathetic control in patients with heart disease. N. Engl. J. Med.

[bib29] Guyton A.C, Harris J.W (1951). Pressoreceptor-autonomic oscillation; a probable cause of vasomotor waves. Am. J. Physiol.

[bib30] Guyton A.C, Coleman T.G, Granger H.J (1972). Circulation: overall regulation. Annu. Rev. Physiol.

[bib31] Hidaka I, Nozaki D, Yamamoto Y (2000). Functional stochastic resonance in the human brain: noise induced sensitization of baroreflex system. Phys. Rev. Lett.

[bib32] Hyndman B.W, Kitney R.I, Sayers B.M (1971). Spontaneous rhythms in physiological control systems. Nature.

[bib36] Iellamo F, Di Rienzo M, Lucini D, Legramante J.M, Pizzinelli P, Castiglioni P, Pigozzi F, Pagani M, Parati G (2006). Muscle metaboreflex contribution to cardiovascular regulation during dynamic exercise in microgravity: insights from mission STS-107 of the space shuttle Columbia. J. Physiol.

[bib37] Julien C (2006). The enigma of Mayer waves: facts and models. Cardiovasc. Res.

[bib33] Karemaker J.M, Wesseling K.H (2008). Variability in cardiovascular control: the baroreflex reconsidered. Cardiovasc. Eng.

[bib34] Kitney R.I (1979). A nonlinear model for studying oscillations in the blood pressure control system. J. Biomed. Eng.

[bib35] Krieger E.M (1986). Neurogenic mechanisms in hypertension: resetting of the baroreceptors. Hypertension.

[bib38] Landgren S (1952). On the excitation mechanism of the carotid baroreceptors. Acta Physiol. Scand.

[bib39] Laude D (2004). Comparison of various techniques used to estimate spontaneous baroreflex sensitivity (the EuroBaVar study). Am. J. Physiol. Regul. Integr. Comp. Physiol.

[bib40] Lohmeier T.E, Irwin E.D, Rossing M.A, Serdar D.J, Kieval R.S (2004). Prolonged activation of the baroreflex produces sustained hypotension. Hypertension.

[bib41] Mancia, G. & Mark, A. L. 1983 Arterial baroreflexes in humans. In *Handbook of physiology, the cardiovascular system IV*, vol. 3 (eds J. T. Shepherd & F. M. Abboud), pp. 755–794. Bethesda, MD: American Physiological Society.

[bib42] Mancia G, Parati G (2003). The role of blood pressure variability in end-organ damage. J. Hypertens. Suppl.

[bib43] Mancia G, Ferrari A.U, Gregorini L, Parati G, Pomidossi G, Zanchetti A (1982). Effects of isometric exercise on the carotid beroreflex in hypertensive subjects. Hypertension.

[bib44] Mancia G, Parati G, Castiglioni P, Di Rienzo M (1999). Effect of sinoaortic denervation on frequency-domain estimates of baroreflex sensitivity in conscious cats. Am. J. Physiol. Heart Circ. Physiol.

[bib45] McCubbin J.W, Green J.H, Page I.H (1956). Baroreceptor function in chronic renal hypertension. Circ. Res.

[bib46] Montano N, Gnecchi-Ruscone T, Porta A, Lombardi F, Malliani A, Barman S.M (1996). Presence of vasomotor and respiratory rhythms in the discharge of single medullary neurons involved in the regulation of cardiovascular system. J. Auton. Nerv. Syst.

[bib47] Mukkamala R, Toska K, Cohen R.J (2003). Noninvasive identification of the total peripheral resistance baroreflex. Am. J. Physiol. Heart Circ. Physiol.

[bib48] Nollo G, Faes L, Porta A, Pellegrini B, Ravelli F, Del Greco M, Disertori M, Antolini R (2002). Evidence of unbalanced regulatory mechanism of heart rate and systolic pressure after acute myocardial infarction. Am. J. Physiol. Heart Circ. Physiol.

[bib49] Nollo G, Faes L, Porta A, Antolini R, Ravelli F (2005). Exploring directionality in spontaneous heart period and systolic pressure variability interactions in humans: implications in the evaluation of baroreflex gain. Am. J. Physiol. Heart Circ. Physiol.

[bib50] Nollo G, Faes L, Antolini R, Porta A (2009). Assessing causality in normal and impaired short-term cardiovascular regulation via nonlinear prediction methods. Phil. Trans. R. Soc. A.

[bib51] Parati G, Di Rienzo M, Bertinieri G, Pomidossi G, Casadei R, Groppelli A, Pedotti A, Zanchetti A, Mancia G (1988). Evaluation of the baroreceptor–heart rate reflex by 24-hour intra-arterial blood pressure monitoring in humans. Hypertension.

[bib52] Parati G, Frattola A, Di Rienzo M, Castiglioni P, Pedotti A, Mancia G (1995a). Effects of aging on 24 hour dynamic baroreceptor control of heart rate in ambulant subjects. Am. J. Physiol. Heart Circ. Physiol.

[bib53] Parati G, Saul J.P, Di Rienzo M, Mancia G (1995b). Spectral analysis of blood pressure and heart rate variability in evaluating cardiovascular regulation: a critical appraisal. Hypertension.

[bib54] Parati G, Di Rienzo M, Mancia G (2000). How to measure baroreflex sensitivity: from the cardiovascular laboratory to daily life. J. Hypertens.

[bib55] Patton D.J, Triedman J.K, Perrott M.H, Vidian A.A, Saul J.P (1996). Baroreflex gain: characterization using autoregressive moving average analysis. Am. J. Physiol. Heart Circ. Physiol.

[bib56] Persson P.B, Ehmke H, Köhler W.W, Kirchheim H.R (1990). Identification of major slow blood pressure oscillations in conscious dogs. Am. J. Physiol. Heart Circ. Physiol.

[bib57] Porta A, Baselli G, Rimoldi O, Malliani A, Pagani M (2000). Assessing baroreflex gain from spontaneous variability in conscious dogs: role of causality and respiration. Am. J. Physiol. Heart Circ. Physiol.

[bib58] Porta A, Furlan R, Rimoldi O, Pagani M, Malliani A, van de Borne P (2002). Quantifying the strength of the linear causal coupling in closed loop interacting cardiovascular variability signals. Biol. Cybern.

[bib59] Potts J.T, Mitchell J.H (1998). Rapid resetting of carotid baroreceptor reflex by afferent input from skeletal muscle receptors. Am. J. Physiol. Heart Circ. Physiol.

[bib60] Radaelli A, Castiglioni P, Centola M, Cesana F, Balestri G, Ferrari A.U, Di Rienzo M (2006a). Adrenergic origin of very low frequency blood pressure oscillations in the unanesthetized rat. Am. J. Physiol. Heart Circ. Physiol.

[bib61] Radaelli A (2006b). Enhanced baroreceptor control of the cardiovascular system by polyunsaturated fatty acids in heart failure patients. J. Am. Coll. Cardiol.

[bib62] Ray C.A (2000). Interaction of the vestibular system and baroreflexes on sympathetic nerve activity in humans. Am. J. Physiol. Heart Circ. Physiol.

[bib63] Ringwood J.V, Malpas S.C (2001). Slow oscillations in blood pressure via a nonlinear feedback model. Am. J. Physiol. Regul. Integr. Comp. Physiol.

[bib64] Seagard J.L, Gallemberg L.A, Hopp F.A, Dean C (1992). Acute resetting in two functionally different types of carotid baroreceptors. Circ. Res.

[bib65] Seidel H, Herzel H (1998). Bifurcations in a nonlinear model of the baroreceptor–cardiac reflex. Physica D.

[bib66] Sleight P, La Rovere M.T, Mortara A, Pinna G, Maestri R, Leuzzi S, Bianchini B, Bernardi L (1995). Physiology and pathophysiology of heart rate and blood pressure variability in humans: is power spectral analysis largely an index of baroreflex gain?. Clin. Sci.

[bib67] Smith H.S, Sleight P, Pickering G.W (1969). Reflex regulation of arterial pressure during sleep in man. A quantitative method of assessing baroreflex sensitivity. Circ. Res.

[bib68] Soma R, Nozaki D, Kwak S, Yamamoto Y (2003). 1/*f* noise outperforms white noise in sensitizing baroreflex function in the human brain. Phys. Rev. Lett.

[bib69] Somers V.K, Mark A.L, Abboud F.M (1991). Interaction of baroreceptor and chemoreceptor reflex control of sympathetic nerve activity in normal humans. J. Clin. Invest.

[bib70] Task Force of the European Society of Cardiology and the North American Society of Pacing and Electrophysiology (1996). Heart rate variability: standards of measurement, physiological interpretation and clinical use. Circulation.

[bib71] TenVoorde B.J, Kingma R (2000). A baroreflex model of short term blood pressure and heart rate variability. Stud. Health Technol. Inform.

[bib72] Thrasher T.N (2002). Unloading arterial baroreceptors causes neurogenic hypertension. Am. J. Physiol. Regul. Integr. Comp. Physiol.

[bib73] Thrasher T.N (2005). Baroreceptors, baroreceptor unloading, and the long-term control of blood pressure. Am. J. Physiol. Regul. Integr. Comp. Physiol.

[bib74] Tozawa M, Iseki K, Yoshi S, Fukiyama K (1999). Blood pressure variability as an adverse prognostic risk factor in end-stage renal disease. Nephrol. Dial. Transplant.

[bib76] Wang H, Ju K, Chon K.H (2007). Closed-loop nonlinear system identification via the vector optimal parameter search algorithm: application to heart rate baroreflex control. Med. Eng. Phys.

[bib77] Wesseling, K. H. & Settels, J. J. 1985 Baromodulation explains short-term blood pressure variability. In *Psychophysiology of cardiovascular control. Models, methods and data*, pp. 69–97. New York, NY: Plenum.

[bib78] Wolf M.M, Varigos G.A, Hunt D, Sloman J.G (1978). Sinus arrhythmia in acute myocardial infarction. Med. J. Aust.

